# Dosage Regulation of the Active X Chromosome in Human Triploid Cells

**DOI:** 10.1371/journal.pgen.1000751

**Published:** 2009-12-04

**Authors:** Xinxian Deng, Di Kim Nguyen, R. Scott Hansen, Daniel L. Van Dyke, Stanley M. Gartler, Christine M. Disteche

**Affiliations:** 1 Department of Pathology, University of Washington, Seattle, Washington, United States of America; 2 Department of Medicine, University of Washington, Seattle, Washington, United States of America; 3 Department of Genome Sciences, University of Washington, Seattle, Washington, United States of America; 4 Mayo Clinic College of Medicine, Rochester, Minnesota, United States of America; Massachusetts General Hospital, Howard Hughes Medical Institute, United States of America

## Abstract

In mammals, dosage compensation is achieved by doubling expression of X-linked genes in both sexes, together with X inactivation in females. Up-regulation of the active X chromosome may be controlled by DNA sequence–based and/or epigenetic mechanisms that double the X output potentially in response to autosomal factor(s). To determine whether X expression is adjusted depending on ploidy, we used expression arrays to compare X-linked and autosomal gene expression in human triploid cells. While the average X:autosome expression ratio was about 1 in normal diploid cells, this ratio was lower (0.81–0.84) in triploid cells with one active X and higher (1.32–1.4) in triploid cells with two active X's. Thus, overall X-linked gene expression in triploid cells does not strictly respond to an autosomal factor, nor is it adjusted to achieve a perfect balance. The unbalanced X:autosome expression ratios that we observed could contribute to the abnormal phenotypes associated with triploidy. Absolute autosomal expression levels per gene copy were similar in triploid versus diploid cells, indicating no apparent global effect on autosomal expression. In triploid cells with two active X's our data support a basic doubling of X-linked gene expression. However, in triploid cells with a single active X, X-linked gene expression is adjusted upward presumably by an epigenetic mechanism that senses the ratio between the number of active X chromosomes and autosomal sets. Such a mechanism may act on a subset of genes whose expression dosage in relation to autosomal expression may be critical. Indeed, we found that there was a range of individual X-linked gene expression in relation to ploidy and that a small subset (∼7%) of genes had expression levels apparently proportional to the number of autosomal sets.

## Author Summary

Many organisms have a single X chromosome in males and two in females, leading to a chromosome imbalance between autosomes and sex chromosomes and between the sexes. In mammals, this dosage imbalance is adjusted by doubling expression of X-linked genes in both sexes and by silencing one X chromosome in females. We used expression array analyses of human triploid cultures to test X chromosome expression in the presence of three sets of autosomes and address the question of an autosomal counting factor. We found that overall X-linked gene expression is not tripled in the presence of three sets of autosomes. However, in triploid cells with a single active X chromosome, its expression is adjusted upward presumably by an epigenetic mechanism that senses the active X/autosome ratio. Based on the range of individual gene expression we identified a subset of dosage-sensitive genes whose expression is apparently proportional to the ploidy. Our findings are important for understanding the regulation of the X chromosome and the role of ploidy in the balance of gene expression and associated phenotypes.

## Introduction

Dosage compensation restores a balanced network of gene expression between autosomes and sex chromosomes in males (XY) and females (XX) [1]. Strategies to achieve this vary among species [2]. In *Drosophila*, a male-specific ribonucleoprotein complex binds to the X chromosome and modifies chromatin structure to increase expression of most X-linked genes by two fold. X up-regulation also occurs in *C. elegans* and in mammals but in both sexes [3],[4]. Silencing of one X chromosome in mammalian females and repression of both X chromosomes in *C. elegans* hermaphrodites have been adapted to avoid hyper-expression in the homogametic sex. While these repressive processes have been well studied, the mechanisms of X up-regulation in mammals and worms remain to be determined.

Human triploids occur in about 1% of conceptions. Depending on their sex chromosome composition (XXX, XXY, XYY), triploid cells show a variety of X inactivation patterns [5]–[8]. Female triploid (XXX) fibroblast clones with either one or two active X chromosomes (Xa) have been successfully established [5]. These cloned lines with a defined number of Xa's as well as XYY triploid cell cultures provide a mean to test X chromosome expression in the presence of three sets of autosomes, which could help understand the underlying mechanisms of X up-regulation and test the potential existence of an autosomal counting factor. If DNA sequence changes that affect promoters, enhancers, and/or regions that alter mRNA elongation and stability were sufficient to mediate the two-fold elevated expression of X-linked genes in normal diploid cells, we would predict that the X:autosome (X:A) expression ratio would be 0.67 (i.e. 2/3) in triploid cells with a single Xa and 1.33 with two Xa's, assuming that autosomal genes have a steady expression level per gene copy and thus produce approximately 1.5-fold more products in triploid versus diploid cells. A second possibility is that epigenetic chromatin remodeling of the X chromosome and/or of the autosomes may modulate the balance of gene expression, in which case the X:A expression ratio in triploid cells with one Xa or two Xa's would differ from 0.67 and 1.33, respectively. If this epigenetic regulation of the active X chromosome in triploid cells was based on a counting mechanism mediated by autosomal factor(s) that would triple the output from the Xa, the predicted X:A expression ratio in triploid cells with one Xa would be 1 (i.e. 3/3), similar to the situation in diploid cells [4], whereas triploid cells with two Xa's would have a ratio of 2 (i.e. 6/3). Alternatively, both genetic and epigenetic mechanisms could be involved in regulation of X up-regulation, in which case the predicted ratios would be further adjusted. A perfect adjustment to a ratio of 1 could possibly be observed if X-linked and/or autosomal gene expression was adjusted in response to the number of active X chromosomes in relation to autosomes. Studies in *Drosophila* have shown that both X-linked and autosomal gene expression can respond to abnormal genotypes [9].

In the present study, we used expression array analyses to determine the global expression of X-linked genes versus autosomal genes in triploid cell cultures with either one or two Xa's. We found no evidence of a global change in absolute autosomal expression levels per gene copy in triploid versus diploid cells. Our results are consistent with a doubling of expression of most X-linked genes in triploid cells with two Xa's. However, X expression was further increased in triploid cells with one Xa, suggesting an epigenetic adjustment in response to the Xa/autosome ratio. The expression of genes with copies on both sex chromosomes and of genes that escape X inactivation was investigated in triploid cells. We determined that XYY triploid cells had a expression deficiency in genes that escape X inactivation compared to XXX triploid cells. We also found that expression of a small subset of X-linked genes apparently responds to the cell ploidy, suggesting that dosage of these genes is critical.

## Results

### X chromosome expression in triploid cells with one or two active X chromosomes

**Figure 1:**
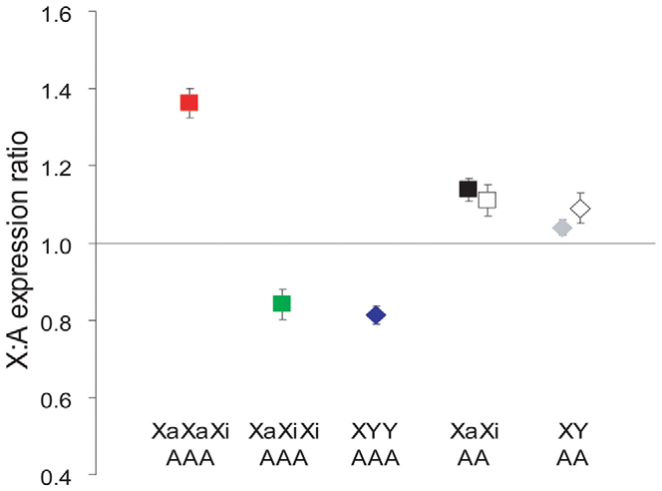
X:autosome expression ratios in triploid cultures. The mean (± standard deviation) X:A expression ratios are shown for each genotype indicated at the bottom (Xa: active X chromosome, Xi: inactive X chromosome, Y: Y chromosome, A: autosome). Three individual triploid fibroblast cultures of each genotype were assayed alongside two female (black square) and two male (gray diamond) diploid fibroblast cultures. The X:A expression ratios calculated from published microarray data for adult fibroblasts from 12 female and 11 male individuals are represented by white square and white diamond, respectively [10].

Six XXX triploid fibroblast clones with either one or two Xa's (XaXiXi or XaXaXi, Xa and Xi being the active and inactive X, respectively) [5], and three XYY triploid fibroblast cultures were analyzed along with two male (XY) and two female (XaXi) control diploid fibroblast cultures using Affymetrix whole genome expression arrays. As expected, while *XIST* expression was absent in XYY cultures, it was two-fold higher in XaXiXi versus XaXaXi cultures, consistent with the number of Xi's (Figure S1) [5]. For each cell line the X:A expression ratio was calculated by dividing the mean expression of 362 X-linked genes (corresponding to 550 probe-sets) by that of 10,267 autosomal genes (corresponding to 16,984 probe-sets) expressed in all cultures. The average X:A expression ratios were 1.04±0.02 and 1.14±0.03, for control male and female diploid fibroblasts, respectively (Figure 1 and Table S1). Analysis of additional published microarray data for 11 male and 12 female adult fibroblasts yielded X:A expression ratios of 1.09±0.04 and 1.11±0.04, respectively, based on 397 X-linked and 8,832 autosomal genes [10]. These analyses are consistent with functional dosage compensation in normal diploid somatic cells and slightly higher X expression in females [4],[11].

XYY and XaXiXi triploid fibroblasts with a single Xa had average X:A expression ratios of 0.81±0.02 and 0.84±0.04, respectively (Figure 1 and Table S1). These X:A expression ratios were significantly above the theoretical value of 0.67 (2/3) (p<0.0005, n = 550, independent one-sample t-test) expected if X expression was strictly doubled while autosomal genes presumably produced 1.5-fold more products in triploid cells, which was confirmed as described below. For this statistical analysis, the expression signal of each of 550 X-linked probe-sets was divided by the mean of autosomal expression to calculate the X:A expression ratio for each X-linked gene in each triploid genotype. For each genotype an independent one-sample t-test was performed to test and reject the null hypothesis that the mean X:A expression ratio was not significantly different from the expected mean (see Materials and Methods). Even when considering that the mean X:A expression ratio was slightly higher than 1 (1.04–1.14) in control diploid cultures the ratio in triploid cells with one Xa remained significantly higher than expected (p = 0.001 and 0.03, respectively, n = 550). Thus, X expression was more than doubled in triploid cultures with one Xa, but it was not tripled since the X:A expression ratios were significantly below the value of 1 expected if expression strictly responded to an autosomal counting factor (p<0.001, n = 550). The average X:A expression ratio in triploid cultures with two active X (XaXaXi) was 1.36±0.04, which was similar to the theoretical value of 1.33 (4/3) expected based on a simple doubling of X expression on active X allele in these cells. In contrast to triploid cells with one Xa, there was no evidence of higher than doubled X expression in cells with two Xa's, i.e. the observed ratio of 1.36 was significantly lower than 1.68 (twice the ratio observed in XaXiXi cells, p = 0.00007, n = 550; Figure 1 and Table S1). We conclude that, on average, expression of X-linked genes is not tripled in triploid cultures; rather, the X chromosome transcriptional output appears to remain basically doubled like in diploid cells, with a further adjustment in triploid cells with a single active X.

### Absolute autosomal expression levels per gene copy in triploid cells

Expression array data must be normalized to genome or autosomal expression in order to be comparable between experiments. To address the possibility that the presence of three sets of autosomes might lead to an adjustment in autosomal rather than X-linked gene expression, we verified that absolute expression levels per gene copy were the same for autosomal genes in triploid and diploid cell cultures by quantifying transcripts per copy number of eight autosomal housekeeping genes. Total nucleic acids (XNA) were prepared from triploid and control diploid cultures under conditions that avoided distortion of the relative ratio of mRNA to DNA [12]. The average fold change in absolute expression levels per gene copy determined by quantitative PCR of the autosomal genes was 0.90±0.35 between triploid and diploid cultures, indicating no adjustment in autosomal expression levels per gene copy relative to ploidy (Figure S2). However, individual gene expression was variable and thus we turned to a global approach to measure absolute gene expression.

**Figure 2:**
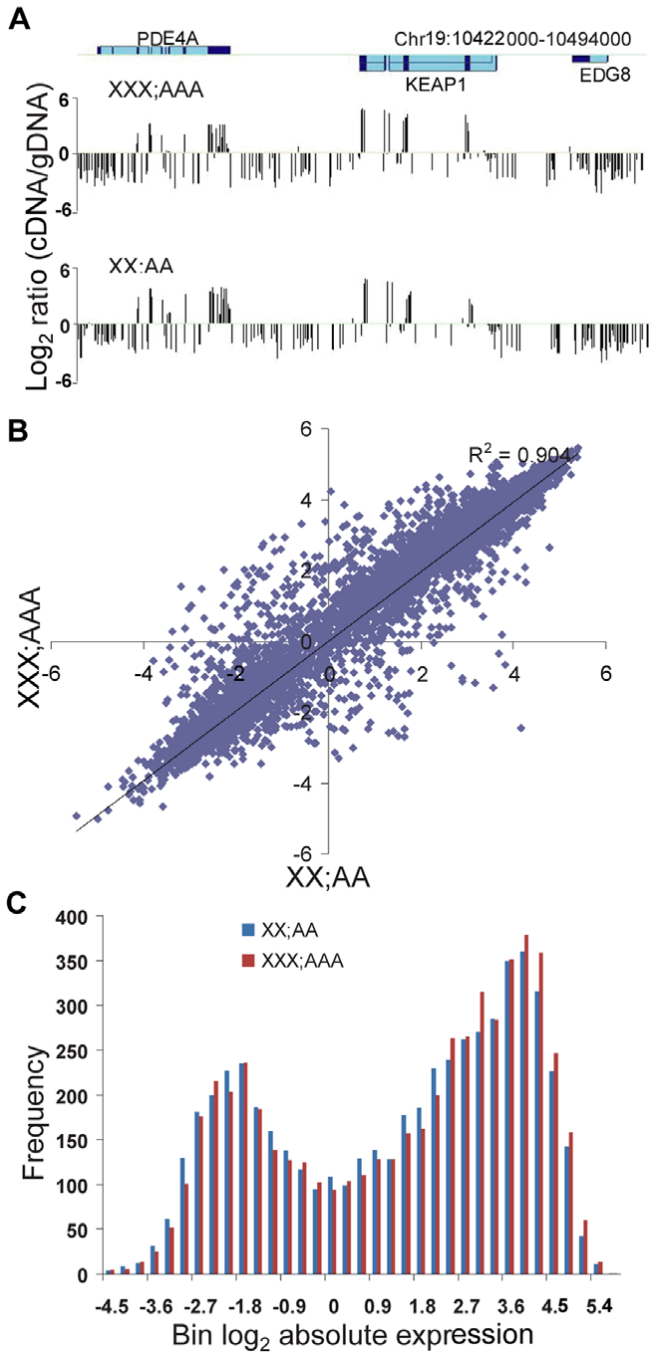
Global absolute autosomal gene expression is similar between triploid and diploid cultures. (A) Example of absolute gene expression levels determined by tiling array hybridization with gDNA and cDNA along a genomic region that includes three autosomal genes (*PDE4A* (phosphodiesterase 4A), *KEAP1* (kelch-like ECH-associated protein 1), and *EDG8* (endothelial differentiation sphingolipid G-protein-coupled receptor 8) in triploid (XXX;AAA) and diploid (XX;AA) cultures. Location of exons (dark blue) and introns (light blue) shown on top are from the UCSC browser [26]. Probes with a high cDNA/gDNA signal ratio are associated with exons, especially at the 3′ end of genes, while probes with a low signal ratio are associated with introns, intergenic regions and low-expressed genes (e.g. *EDG8*). (B) Scatter plot of absolute expression values for 5,473 autosomal genes in triploid XXX;AAA versus diploid XX;AA cultures. The trend had a high R^2^ value (0.90), indicating a highly correlated expression between triploid and diploid cultures. (C) Distributions of absolute expression of autosomal genes show a similar pattern in triploid and diploid cultures. Absolute expression levels were transformed into log_2_ and binned before graphing the data (see also Figure S4).

To compare global absolute expression levels per gene copy NimbleGen DNA tiling arrays were hybridized with Cy3-labeled gDNA and Cy5-labeled cDNA from triploid or diploid XNA. To obtain sufficient materials XNA was prepared from an uncloned XXX triploid culture in which the proportion of cells with one Xa or two Xa's was determined by RNA- and DNA-FISH using a probe for *XIST* to mark the inactive X (Figure S3). The X:A expression ratio calculated from tiling array data was 0.98 (compared to 0.99 for the diploid culture), as predicted based on a mixture of 57% XaXiXi (ratio = 0.84) and 38% XaXaXi cells (ratio = 1.36) (Figure S3). As expected, probes with a high cDNA/gDNA signal ratio after hybridization to the tiling array were associated with exons, especially at the 3′ end of genes (Figure 2A). Scatter plots of absolute expression values in log_2_ scale for 5,473 autosomal genes showed similar profiles between triploid and diploid cultures, with a high R-square value (0.90) (Figure 2B); this concordance was confirmed by calculating an overall 1.07 fold change between average absolute expression levels per gene copy of 5,473 autosomal genes in triploid and diploid cultures. Although the distributions of absolute expression levels in log_2_ scale were similar between cultures, they were unexpectedly bimodal. We investigated them by separating genes into two groups: those with a level <1 (group 1: 1,682 genes) or ≥1 (group 2: 3,478 genes) (Figure 2C). Within each group, the absolute autosomal expression levels had a normal distribution and the fold change between triploid and diploid cultures remained 1.07 (Figure S4A, S4B). Increases in total exon length, length of the last exon, and number of exons for genes in group 2 versus group 1 probably contributed to a shift to higher signals and an apparently bimodal distribution (Figure S4C, S4D, S4E). We conclude that the absolute level of autosomal gene expression, i.e., expression level per gene copy, does not apparently differ between triploid and diploid cells. This suggests that expression adjustment of the X chromosome but not autosomes prevails in triploid cell cultures.

### X-linked gene distribution of expression in triploid cultures

**Figure 3:**
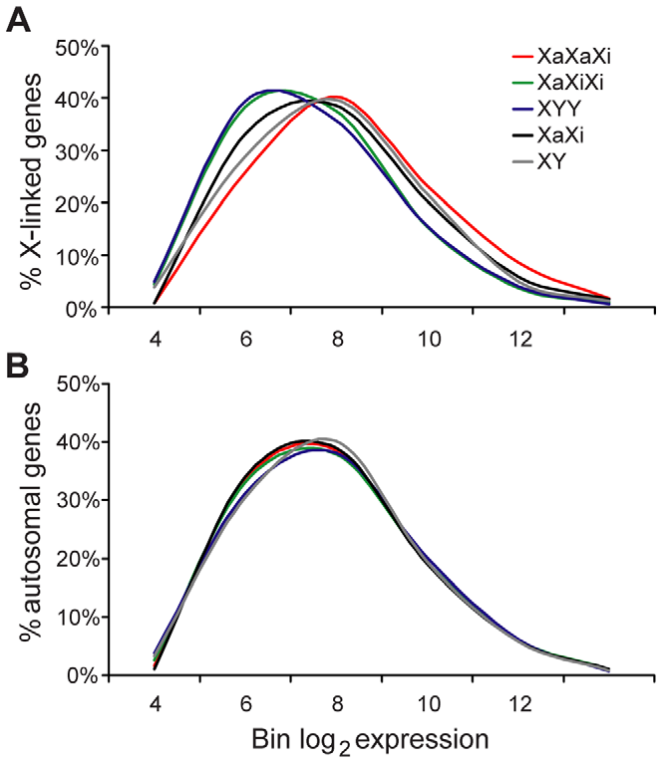
Distributions of X-linked and autosomal gene expression in human triploid cultures. (A) Normalized distribution curves representing expression of 362 X-linked genes differ between triploid and diploid cultures. The average distribution curve for three XaXaXi cultures is shifted to the right (higher values) compared to the average for two female (XaXi) diploid cultures, whereas the average curves for three XaXiXi cultures and three XYY cultures are both shifted left compared to the average for two female (XaXi) and two male (XY) diploid cultures. (B) Normalized distribution curves representing expression of 10,267 autosomal genes are similar between triploid and diploid cultures. Normalized expression levels were transformed into log_2_ and binned before graphing the data. The distribution curves were smoothed.

**Figure 4:**
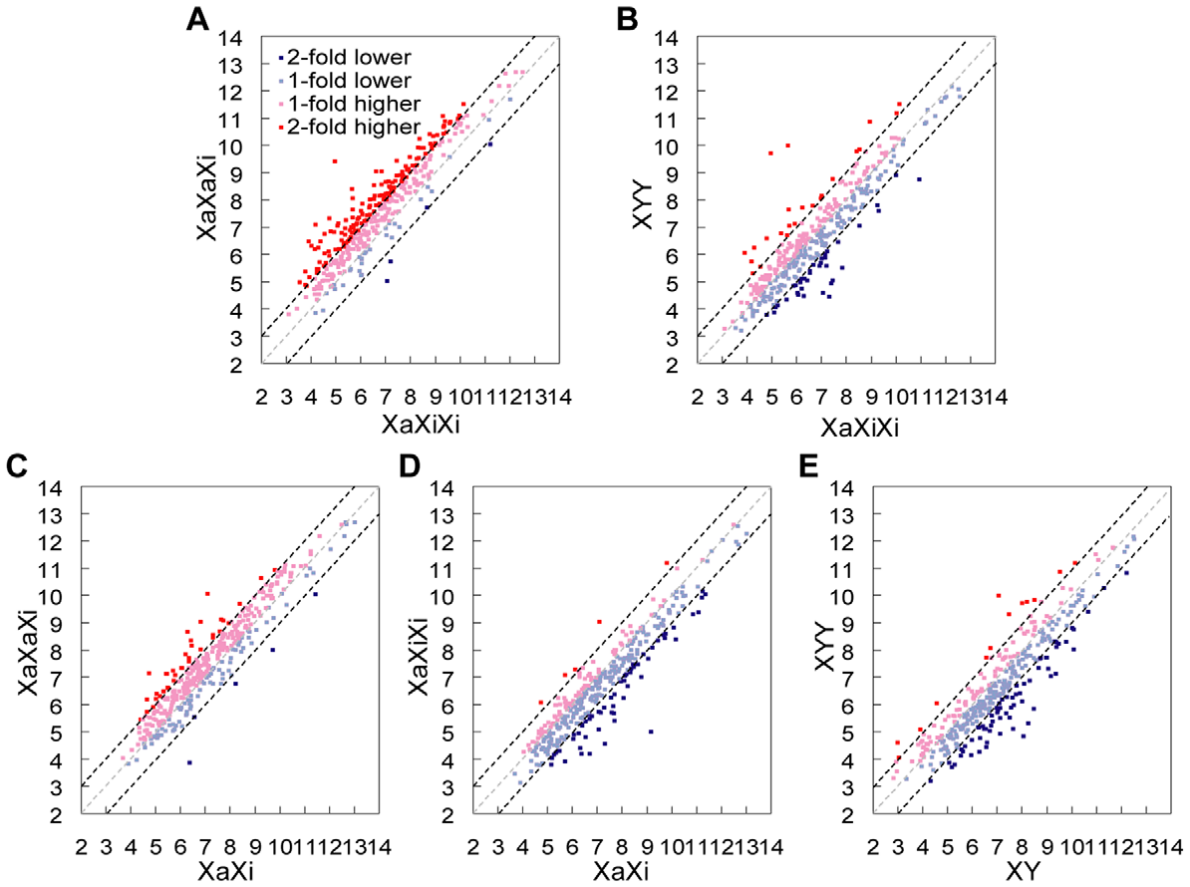
Expression of individual X-linked genes in human triploid and diploid cultures. Scatter plots of X-linked gene expression normalized to autosomal expression (in log_2_ scale). Red symbols indicate genes with more than two-fold higher expression, pink symbols, genes with one- to two-fold higher expression, light blue symbols, genes with one- to two-fold lower expression and blue symbols, genes with more than two-fold lower expression. The dotted lines represent two-fold (black), one-fold (gray) and 0.5-fold (black) cutoffs, respectively. (A) Higher expression of X-linked genes in XaXaXi versus XaXiXi cultures. (B) Similar expression of X-linked genes in XYY versus XaXiXi cultures. (C) Higher expression of X-linked genes in XaXaXi versus XaXi cultures. (D) Lower expression of X-linked genes in XaXiXi versus XaXi cultures. (E) Lower expression of X-linked genes in XYY versus XY cultures.

Based on expression array data (after normalization to autosomal expression) the distributions of expression of X-linked genes differed between triploid and diploid cultures (Figure 3). Compared to diploid cultures, triploid cultures with one Xa showed a clear shift towards lower expression values for X-linked genes (single factor ANOVA test p = 0.001 and 0.0003 for XaXiXi and XYY, respectively; Figure 3A). In contrast, triploid cultures with two Xa's showed a shift towards higher expression values for X-linked genes (single factor ANOVA test p = 0.0002). Comparisons of individual gene expression levels (normalized to autosomal expression) between triploid cultures with one or two Xa's using scatter plots showed that most X-linked genes (307/362 or 85%; spots above a central diagonal in Figure 4A) had higher expression in cultures with two Xa's versus one Xa. A subset of X-linked genes (38%; red spots in Figure 4A) had an increase of 2-fold or higher, suggesting that their expression was proportional to the number of Xa's in triploid cultures. However, the rest of genes (62%; Figure 4A) did not show this two-fold increase in cultures with two Xa's versus one Xa, consistent with lower expression from each Xa in XaXaXi cultures compared to expression from the single Xa in XaXiXi cultures.

**Figure 5:**
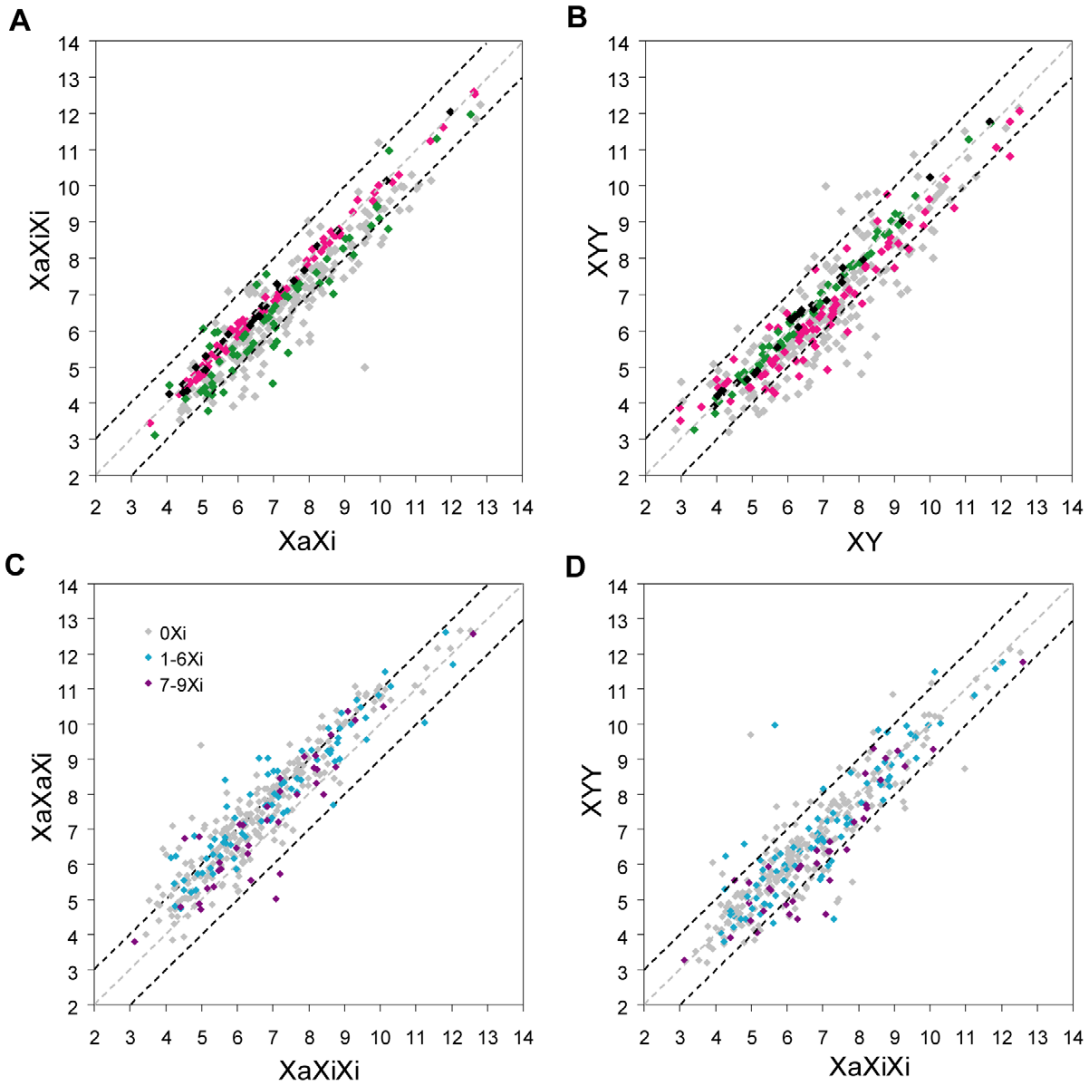
Identification of dosage-sensitive X-linked genes and effects of the X inactive status on gene expression. Scatter plots of X-linked gene expression normalized to autosomal expression (in log_2_ scale). (A) XaXiXi versus XaXi. (B) XYY versus XY. (C) XaXaXi versus XaXiXi. (D) XYY versus XaXiXi. (A,B) X-linked genes with similar expression (<1.2 fold change, *p*>0.2) in triploid (XaXiXi or XYY) versus diploid cultures are highlighted in (A,B): pink symbols: 82 X-linked genes with similar expression in XaXiXi versus XaXi; green symbols: 62 X-linked genes with similar expression in XYY versus XY; black symbols: 26 X-linked genes with similar expression both in XaXiXi versus XaXi and in XYY versus XY; gray symbols: 192 X-linked genes with no evidence of similar expression either in XaXiXi versus XaXi or in XYY versus XY. (C,D) X-linked genes that escape X inactivation are highlighted in (C,D). The escape status of X-linked genes were obtained from a previous study [14]: gray symbols (0Xi): X-linked genes subject to X inactivation (not expressed in rodent x human hybrid cells that retain an inactive human X); light blue symbols (1-6Xi): X-linked genes with variable escape (expressed in 1-6/9 hybrid lines); purple symbols (7-9Xi): X-linked genes that consistently escape (expressed in 7-9/9 hybrid lines). The dotted lines represent two-fold (black), one-fold (gray) and 0.5-fold (black) cutoffs, respectively.

Scatter plots of X-linked gene expression levels (normalized to autosomal expression) to compare sex-matched triploid to diploid cultures showed a consistent proportion (69–73%) of X-linked genes with higher cumulative expression relative to autosomes in all three XaXaXi cultures compared to XaXi cultures (Figure 4C and Figure S5A, S5B, S5C). The proportion of X-linked genes with lower expression relative to autosomes in all three XaXiXi cultures versus XaXi cultures and all three XYY cultures versus XY cultures was 71–81% and 68–71%, respectively (Figure 4D and 4E, Figure S5D, S5E, S5F, S5G, S5H, S5I). In both of these sex-matched comparisons a subset of genes (30% and 24%, respectively) was identified as having similar expression levels relative to autosomes in triploid and diploid cells (<1.2-fold change, p>0.2, two-tail unpaired student t-test; Figure 5A and 5B, Table 1, and Table S2). This subset of genes, whose expression was nearly tripled in triploid cultures with a single Xa, could represent genes for which a balanced expression was critical. Twenty-six such genes were identified in both female and male triploid cultures (black spots in Figure 5A and 5B and Table 1 and Table S2). These genes consistently showed similar expression levels relative to autosomes, with average fold changes close to 1 between triploid cultures and between triploid and diploid cultures, suggesting that they are regulated in a dosage-sensitive manner (Table 1). Their map position revealed a slightly more prevalent location on the short arm of the human X chromosome (2.2 genes/10 Mb) compared to the long arm (1.4 genes/10 Mb) (Table S2). GO analysis showed no specific functional classification [13].

**Table 1:** Average fold change in expression of X-linked genes grouped either by sex-matched triploid/diploid comparisons or by X inactivation status.

Group		Number of X-linked genes	Average fold change^a^					
			XaXaXi/XaXiXi^b^	XaXaXi/XaXi	XaXiXi/XYY	XaXiXi/XaXi	XYY/XY	XaXi/XY
Grouped by triploid/diploid expression comparisons	1^c^	108	1.2	1.14	1.31	0.95	0.71	0.99
	2^d^	88	1.43	1.12	1.09	0.78	1.03	1.43
	3^e^	26	1.08	1.08	1.08	1.01	1.04	1.12
Grouped by X inactivation status	4^f^	161	1.63	1.22	1.17	0.75	0.73	1.14
	5^g^	80	1.55	1.09	0.97	0.70	0.80	1.11
	6^h^	37	1.27	1.09	1.46	0.86	0.72	1.12
Total^i^		362	1.57	1.17	1.08	0.74	0.79	1.15

a Average expression fold change between cultures after normalization to autosomal gene expression

b Xa: active X chromosome, Xi: inactive X chromosome

c Group 1: genes with similar expression in XaXiXi versus XaXi

d Group 2: genes with similar expression in XYY versus XY

e Group 3: genes that overlap between groups 1 and 2, i.e. have similar expression in both XaXiXi versus XaXi and XYY versus XY comparisons.

f Group 4: genes subject to X inactivation (not expressed in rodent x human hybrid cell lines that retain an inactive human X [14].

g Group 5: genes that variably escape from X inactivation (expressed in 1-6/9 hybrid lines) [14].

h Group 6: genes that consistently escape from X inactivation (expressed in 7-9/9 hybrid lines) [14]. PAR1 genes were not included.

i All 362 X-linked genes detected by microarray expression analysis in all diploid and triploid cell cultures.

### Expression of PAR1 and of genes that escape X inactivation in triploid cultures

**Figure 6:**
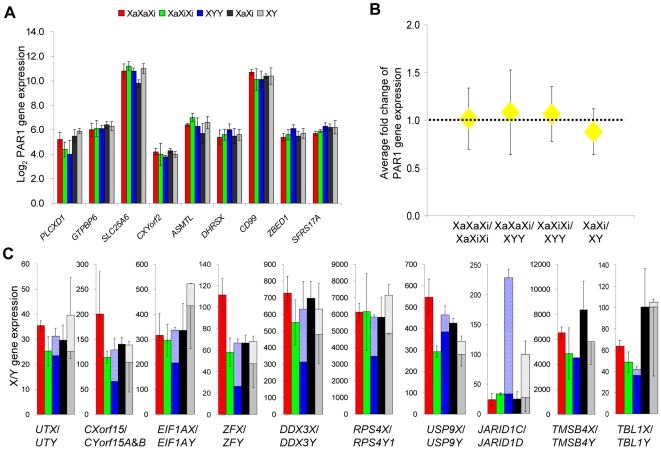
Expression of PAR1 genes and non-pseudoautosomal X/Y gene pairs in triploid and diploid cultures. (A,B) Expression of PAR1 genes is similar between triploid and diploid cultures. (A) Average expression levels (± standard deviation) normalized to autosomal gene expression are shown in log_2_ for nine PAR1genes [*PLCXD1*: phosphatidylinositol-specific phospholipase C, X domain containing 1; *GTPBP6*: GTP binding protein 6; *SLC25A6*:solute carrier family 25 (mitochondrial carrier; adenine nucleotide translocator), member 6; *CXYorf2*: chromosome X and Y open reading frame 2; *ASMTL*: acetylserotonin O-methyltransferase-like; *DHRSX*: dehydrogenase/reductase (SDR family) X-linked; *CD99*: CD99 molecule; *ZBED1*: zinc finger, BED-type containing 1; *SFRS17A*: splicing factor, arginine/serine-rich 17A] in XaXaXi, XaXiXi, XYY triploid cultures, and in XaXi and XY diploid controls. (B) Average expression fold changes between triploid cultures with one or two Xa's and between female and male diploid cultures for nine PAR1 genes were not significantly different from 1.0 (p = 0.43, 0.29, 0.24, and 0.07 respectively; independent one-sample t-test, n = 9). (C) Cumulative expression of X- and Y-linked paralogs of XY gene pairs in human triploid and diploid cultures. Expression of the X- and Y-linked genes was normalized to autosomal gene expression. Stacked bars show the cumulative expression of X (solid bar) and Y paralogs (striped bar) in XaXaXi, XaXiXi, XYY triploid cultures, and XaXi and XY diploid controls. The X-linked paralogs are shown with (+) and (−) error bars in XXX and XX cell cultures and only with (−) error bars in XYY and XY cell cultures. The Y-linked paralogs are shown with (+) error bars. *UTX*/*UTY*: ubiquitously transcribed tetratricopeptide repeat, X chromosome/Y-linked; *CXorf15*/*CYorf15A* & *B*: chromosome X open reading frame 15/chromosome Y open reading frame 15A & chromosome Y open reading frame 15B; *EIF1AX*/*EIF1AY*: eukaryotic translation initiation factor 1A, X-linked/Y-linked; *ZFX*/*ZFY*: zinc finger protein, X-linked/Y-linked; *DDX3X*/*DDX3Y*: DEAD (Asp-Glu-Ala-Asp) box polypeptide 3, X-linked/Y-linked; *RPS4X/RPS4Y1*:ribosomal protein S4, X-linked/Y-linked 1; *USP9X*/*USP9Y*: ubiquitin specific peptidase 9, X-linked/Y-linked; *JARID1C*/*JARID1D*: jumonji, AT rich interactive domain 1C/jumonji, AT rich interactive domain 1D; *TMSB4X*/*TMSB4Y*: thymosin beta 4, X-linked/Y-linked; *TBL1X*/*TBL1Y*: transducin (beta)-like 1X-linked/Y-linked.

Specific categories of X-linked genes such as genes located within the pseudoautosomal region (PAR) are not subject to dosage compensation mechanisms and thus were used as controls in our study. Analyses of nine PAR1 genes showed similar expression levels between triploid cultures. The average fold changes were 1.02 (XaXaXi/XaXiXi), 1.08 (XaXaXi/XYY), and 1.07 (XaXiXi/XYY), indicating that PAR1 genes behave like autosomal genes, independent of the sex complement and of the number of Xa's (Figure 6A and 6B). This supports the notion that PAR1 genes escape from X inactivation [14], and are not up-regulated on the active X (Nguyen and Disteche, unpublished data).

Expression of non-pseudoautosomal X/Y gene pairs, in which the X paralogs escaped X inactivation [15],[16], was also examined to determine whether Y expression and/or escape from X inactivation could help balance expression in triploid cultures (Figure 6C). For 8/10 pairs (*UTX*/*UTY*, *CXorf15*/*CYorf15A* & *B*, *EIF1AX*/*EIF1AY, ZFX*/*ZFY*, *DDX3X*/*DDX3Y, RPS4X/RPS4Y1, USP9X/USP9Y, JARID1C/JARID1D)* significant Y expression was detected that was not due to cross-hybridization with the X paralog (Table S1). Cumulative expression of most X and Y paralogs in XYY cultures was not significantly different from total X expression in XaXiXi cultures (Figure 6C), suggesting that Y expression could potentially help compensate, would the paralogs provide similar functions as is the case for some gene pairs [17], but not others [18],[19]. An exceptional Y-linked gene was *JARID1D*, which had very high expression compared to *JARID1C*. For most X paralogs, cumulative expression was generally lower in XaXiXi versus XaXaXi cultures, consistent with lower expression from alleles on the Xi (Figure 6C) [14].

To examine the effects of escape from X inactivation on the expression of genes in triploid cultures non-pseudoautosomal X-linked genes (with or without a Y paralog) were grouped into three categories based on a previous survey [14]: (1) genes subject to X inactivation (not expressed in rodent x human hybrid cells that retain an inactive human X, group 4 in Table 1), (2) genes with variable escape (expressed in 1-6/9 hybrid lines, group 5 in Table 1), and (3) genes that consistently escape (expressed in 7-9/9 hybrid lines, group 6 in Table 1) [14]. Predictably, the expression fold change between XaXaXi and XaXiXi cultures was the lowest for genes that escape X inactivation as expected for genes whose expression depends on the total number of X chromosomes (Figure 5C and Table 1). However, this fold change was higher than 1, consistent with lower expression from alleles on the Xi. Genes that consistently escape X inactivation had higher expression in XaXiXi versus XYY cultures compared to genes subject to X inactivation, (Figure 5D and Table 1). We conclude that XYY triploid cultures have a deficiency in expression of escape genes compared to XXX triploid cultures with one or two active X chromosomes.

## Discussion

Our results indicate that X-linked gene expression is up-regulated approximately two-fold in triploid cultures with two active X chromosomes like in diploid cultures [3],[4]. However, in triploid cultures with a single active X, X expression is apparently adjusted further upward, although not tripled. Thus, global X-linked gene expression does not strictly respond to an autosomal counting factor in triploid cells. However, comparisons between triploid and diploid cultures showed a range of individual gene expression, suggesting that not all genes respond to a change in ploidy in the same manner. We previously proposed two mechanisms for X up-regulation [4]: (1) permanent DNA sequence changes during evolution that may affect promoters, enhancers, and/or regions that alter mRNA elongation and stability; (2) epigenetic mechanisms that may control X expression similar to the situation in *Drosophila* [1]. A basic doubling of X expression in triploid cells with two Xa's could be mediated by DNA-sequence modifications and/or by epigenetic mechanisms that normally operate in diploid cells. The notion that the doubling cannot default even when X expression is too high in cells with two active X chromosomes strongly suggests that DNA sequence changes or highly stable epigenetic modifications are involved in X up-regulation. We found no evidence of a global change in absolute expression level per autosomal gene copy in triploid versus diploid cultures, suggesting that gene expression is strictly proportional to the number of autosomal sets. Hence, adjustments in gene expression in triploid cultures mostly affect X-linked genes.

In triploid cells with one Xa, X expression is adjusted upward from a basic doubling. This further adjustment is presumably mediated by an epigenetic mechanism that senses X expression but does not respond to the simple X:A genomic ratio since this occurs both in XYY and XaXiXi cultures. Our data suggest that such a mechanism may act on a subset of genes (∼7% of X-linked genes) whose expression dosage in relation to autosomal expression may be critical. Preliminary investigation in their function did not reveal a specific role for these genes. It is also possible that the triploid cultures with one Xa we examined survived because X expression was adjusted upward, thus reflecting a bias of ascertainment. Interestingly, a previous study in *Drosophila* showed that the normalized enzymatic activity of a protein encoded by an X-linked gene was 0.87 in triploid flies with a single X relative to diploid flies, which suggests a more than doubled, but not tripled expression, similar to our results in human [20].

Previous studies have suggested that fold-changes in expression are often considerably less than differences in gene copy number [3]. It should be noted that we measured steady-state RNA and thus cannot rule out adjustments in protein levels at translation. We previously reported low X:A expression ratios (0.84–0.89) in a subset of normal human diploid tissues [4], suggesting that ratios within this low range could be tolerated in a tissue-specific manner. Conversely, undifferentiated mouse female ES cells with two Xa's have an X:A expression ratio of 1.39 [21], indicating that such a high ratio is tolerated in normal diploid cells during a short time window prior to X inactivation in blastocysts. However, later stage mouse embryos with two Xa's due to failure of X inactivation have a severely abnormal phenotype similar to that of embryos with tetrasomy for an autosome [22]. A previous study of female mouse ES cells with two active X chromosomes showed genome-wide DNA methylation changes in repeated DNA sequences, perhaps in response to the high X expression [23]. However, in our study we did not detect an adjustment in global autosomal expression in triploid cells.

Triploidy, which predominantly results from dispermy, causes lethality in human embryos due to multiple phenotypic abnormalities [8]. The unbalanced X:A expression ratios that we observed could contribute to this lethality. XYY fetuses are much rarer than XXY or XXX fetuses, in which the majority of cells often have two Xa's [5],[24]. We found that XYY cultures had a deficiency in expression of genes that escape X inactivation compared to XaXiXi cultures, which could contribute to the early lethality of XYY triploid fetuses. Relevant to this observation is the prevalent lethality in Turner syndrome fetuses with a single X chromosome, associated with haplo-insufficiency of genes that escape X inactivation. In XYY triploid fetus, Y paralog expression could partially compensate, provided that the function of the X and Y paralogs are similar.

Deficiency in gene expression due to chromosomal abnormalities such as monosomy is less well tolerated than trisomy. Thus, lower than normal X expression may be less compatible with embryonic development than higher expression. A recent study of tetraploid mouse ES cells with a different number of Xa's also supports the notion that an excess of Xa's is more favorable to cell survival than a deficiency [25]. Live-born triploid fetuses have a mixture of cells with one or two Xa's, while early aborted fetuses tend to have a majority of cells with two Xa's [5],[24]. Interestingly, the uncloned culture we studied that consisted of a mixture of cells with one or two Xa's had a balanced overall X:A expression ratio close to 1.0. Thus, mosaicism for cells with different X inactivation patterns may help achieve a more balanced proteome, excluding cell-autonomous protein products. Mechanisms involved in X up-regulation in mammals will need to be investigated at early embryonic stages to follow changes in the level of X-linked gene expression during normal development.

## Materials and Methods

### Cell cultures

The triploid fibroblast cultures consisted of six XXX clones with previously defined X inactivation patterns (75-29-H2, GM04939-2X-1, GM04939-2X-2, 75-29-E4, 75-29-F3 and 75-29-F9) [5], two XXX uncloned cultures (69,XXXCD2 and 69,XXXCD3), three XYY cultures (69,XYY1, 69,XYY2, 69,XYY4), two male and two female diploid cultures, all derived from aborted fetuses and previously characterized by cytogenetic analysis (Table S1). For 69,XXXCD2 we carried out X enumeration using FISH with CEPX alpha satellite and X inactivation analysis using RNA FISH for *XIST* (X inactive-specific transcript) using probes from Vysis (Downers Grove, IL) and standard protocols. The study was approved by the IRB at the University of Washington and at the Mayo Clinic College of Medicine.

### Expression array analysis

Total RNA was prepared using RNeasy kits (Qiagen) with on-column DNaseI digestion prior to quality assay, labeling, and hybridization to Affymetrix human HG-U133 2.0 plus chip (Santa Clara, CA). Array hybridizations were done at the Microarray Center (University of Washington, Seattle WA). The raw data files (DAT file) were analyzed by the Affymetrix software (GCOS 1.4) to produce the data in .CHP format (Excel). Expressed “probe-sets” were selected as those showing signals above background levels, which were determined by the mean signal value of Y-linked probe-sets identified as absent using Affymetrix MAS Present/Absent calls based on a PM-MM (Perfect match-Mismatch) mode (Table S1). The mean X:A expression ratios calculated for 16,984 expressed autosomal probe-sets (corresponding to 10,267 autosomal genes) and 550 X-linked probe-sets (corresponding to 362 X-linked genes) were similar to ratios obtained using a method previously described (Table S1) [4]. For statistical analysis the signal value of each X-linked probe-set was divided by the autosomal array mean and averaged from three arrays for each triploid genotype to calculate one X:A expression ratio for each X-linked probe-set for each triploid genotype. Independent one-sample t-test (Hypothesis test) was then used to compare the mean of X:A expression ratios in one genotype to expected values. The X:A expression ratios (log_2_ scale) had a normal distribution and thus were used to calculate *z* scores and corresponding *p*-values (*z* ^2^ = (log_2_M–log_2_E)/(s^2^/n) (s: standard deviation; n: number of probe-sets) [21]. Gene expression values were normalized to the median value of expressed autosomal probe-sets for each array to compare profiles (distribution and scatter plots) between cell types. Single factor ANOVA test was used to compare distribution profiles. Expression of individual genes was calculated as the mean value of all normalized probe-sets for a given gene. Two-tail unpaired student t-test was used to evaluate the significance of expression changes of individual genes between different triploid cultures and between triploid and diploid cultures. The microarray data are available in NCBI's Gene Expression Omnibus under GEO Series accession number GSE18877 (http://www.ncbi.nlm.nih.gov/geo/query/acc.cgi?acc=GSE18877).

### Quantitative real-time PCR

Total nucleic acids (XNA, i.e. DNA and RNA) were isolated [12]. Two µg of XNA was reverse-transcribed using random hexamers and SuperScript II reverse transcriptase (Invitrogen). The intergenic region ∼3.5kb upstream of *ACTG1* (actin, gamma 1) was used as the internal reference for gene copy normalization. cDNA-specific primers designed to span large intron(s) of eight autosomal housekeeping genes abundantly and similarly expressed in triploid and diploid cultures were tested for PCR efficiency and R^2^ (square of correlation coefficient) using standard curves (Table S3). After quantitative PCR absolute gene expression was calculated as the ratio between cDNA signals and genomic DNA signals.

### Tiling array analysis of absolute expression

Three µg of XNA from diploid (46;XX4) or triploid cells (69;XXXCD2) was fragmented by sonication (100–600bp), digested by RNase A (Promega), and purified (Qiagen PCR purification kit). Genomic DNA was Cy3- labeled using a standard NimbleGen (Madison, WI) sample labeling protocol. 25 µg of XNA from the same preparation was completely digested by DNaseI (Qiagen) and treated with an RNeasy kit (Qiagen). cDNA was produced using oligo-dT and a Superscript double-strand cDNA Synthesis kit (Invitrogen). The cDNA was Cy5-labeled and co-hybridized with Cy3-labeled gDNA to NimbleGen human HG18 economy tiling array set4 that contains 2.1M probes (50–75 mer; median probe spacing: 205 bp) and covers chromosomes 14–22, X and Y.

Array hybridization and scanning were done at the Genome Resources Center (Fred Hutchinson Cancer Research Center, Seattle, WA). The raw data files (pair file) were processed by NimbleScan software using a CGH algorithm without any normalization between two channels. The probes were mapped to exons according NimbleGen annotation files based on build HG18 (UCSC Genome browser) [26]. The absolute expression level for a given gene was calculated by averaging cDNA/gDNA ratios of five exon-associated probes in the 3′ end of that gene.

## Supporting Information

Figure S1 *XIST* is highly expressed in XXX human triploid fibroblasts but is not expressed in XYY triploid fibroblasts. Expression of *XIST* is ∼2-fold higher in XaXiXi versus XaXaXi cultures (p = 0.02, student's t-test). Three independent cultures were analyzed for each genotype by expression microarrays. Averages and standard deviations are shown.
(0.04 MB PDF)

Figure S2 Absolute expression levels of eight autosomal genes are similar in triploid and diploid cultures. The absolute expression level of each gene in triploid cell cultures is represented as the ratio between absolute expression levels in triploid and diploid cultures (mean±standard deviation). The mean fold change between triploid and diploid cultures was 0.90±0.35. Two triploid cultures were analyzed against four female diploid cultures.FN1: fibronectin 1; SEPT2: septin 2; ARF4: ADP-ribosylation factor 4; YWHAQ: tyrosine 3-monooxygenase/tryptophan 5-monooxygenase activation protein, theta polypeptide; MRLC2: myosin regulatory light chain; DAD1: defender against cell death 1; ARL6IP5: ADP-ribosylation-like factor 6 interacting protein 5; HIF1A: hypoxia-inducible factor 1, alpha subunit.
(0.04 MB PDF)

Figure S3 Characterization of the XXXCD2 triploid fibroblasts. (A) Left two panels: examples of DAPI-stained interphase nuclei in which the number of X chromosomes was determined using DNA FISH with an X-linked alpha centrometric (CEP X) probe. Right panel: percentage of 216 nuclei with 0, 1, 2, or 3 signals. (B) Left two panels: examples of DAPI-stained interphase nuclei in which the number of inactive X chromosomes (Xi) was determined by *XIST* RNA FISH. One nuclei has a single *XIST* RNA signal and the other has two signals (arrows). Right panel: percentage of 296 nuclei with 0, 1, 2, or 3 *XIST* RNA signals.
(0.08 MB PDF)

Figure S4 Analyses of autosomal genes showing bimodal expression distribution detected on tiling arrays. Genes were grouped according to their absolute expression level (cDNA/gDNA): <1 (group 1, 1682 genes); ≥1 (group 2, 3,478 genes). (A,B) Distributions of absolute expression of group 1 genes (A) and group 2 genes (B) were similar in diploid (blue bar) and triploid cells (red bar). Absolute expression levels were calculated by averaging cDNA/gDNA ratios (CGH algorithm) of five exon-associated probes at the 3′ end of each gene. Levels were transformed into log2 and binned before graphing the data. (C-E) Compared to group 1 genes (gray bar), group 2 genes (black bar) have longer total-exon-length (C), longer last-exon (D) and more exons per gene (E). Distributions of frequencies of each feature are shown after transformation in log_10_ and binning.
(0.05 MB PDF)

Figure S5 Consistent expression of X-linked genes in individual triploid versus diploid cultures. (A-C) Expression of 69–73% X-linked genes was consistently higher in three individual XaXaXi triploid cultures (GM1X, GM2X, 7529H2) compared diploid XaXi cultures. For comparison the same genes labeled in (A) were also labeled with the same color in (B,C). (D–F) Expression of 71–81% X-linked genes was consistently lower in three individual XaXiXi triploid cultures (F3, F9, E4) versus diploid cultures (XaXi). For comparison the same genes labeled in D were also labeled with the same color in (E,F). (G–I) Expression of 68–71% X-linked genes was lower in three individual XYY triploid cultures (2, 1a, 4) versus diploid XY cultures. For comparison the same genes labeled in (G) were also labeled with the same color in (H) and (I). Scatter plots show expression values (in log_2_ scale) for 362 X-linked genes after normalized to autosomal gene expression. Pink symbols indicate genes with higher expression and light blue symbols, lower expression. The dotted lines represent two-fold (black), one-fold (gray), and 0.5-fold (black) cutoffs, respectively.
(0.14 MB PDF)

Table S1 Gene expression in human triploid and diploid fibroblast cultures based on microarray analyses. ^a^ Xa: active X chromosome; Xi: inactive X chromosome. ^b^ Almost all 111 Y-linked probe-sets included in the human U133 2.0+ Affymetrix arrays showed an Affymetrix “absent call” in female fibroblasts, suggesting no obvious cross-hybridization. In contrast, about 20% of these probe-sets showed a “present call” in male fibroblasts, indicating expression of some Y-linked genes. ^c^ The mean signal value for Y-linked probe-sets with Affymetrix “absent call” was used as background level to filter out unexpressed genes on each array. ^d^ X:A expression ratios calculated from 16,984 autosomal and 550 X-linked expressed probe-sets for each array. ^e^ X:A expression ratios calculated using a previously described method [3].
(0.03 MB DOC)

Table S2 Note: the expression level of each X-linked gene normalized to the median value of autosomal gene expression from each array was used to calculate fold changes between triploid and diploid cultures. Genes with similar expression (<1.2 fold change, p>0.2) in XaXiXi versus XaXi or in XYY versus XY are listed. Pink symbols indicate genes with similar expression in XaXiXi versus XaXi but not in in XYY versus XY. Green symbols indicate genes with similar expression in XYY versus XY but not in XaXiXi versus XaXi. Black symbols indicate genes with similar expression in both comparisons. In each group, genes were sorted according to their chromosomal location from Affymetrix MM8 annotation. p: X short arm. q: X long arm. XAR: X added region. XCR: X conserved region.
(0.13 MB XLS)

Table S3 Primers used for quantitative PCR assays of autosomal genes. ^a^ The primer position in the transcript follows the gene symbol (F, forward; R, reverse). ^b^ The PCR efficiency and the square of the correlation coefficient (R^2^) were derived from standard curves.
(0.03 MB DOC)

## References

[1] Straub T, Becker PB (2007). Dosage compensation: the beginning and end of generalization.. Nat Rev Genet.

[2] Birchler JA, Fernandez HR, Kavi HH (2006). Commonalities in compensation.. Bioessays.

[3] Gupta V, Parisi M, Sturgill D, Nuttall R, Doctolero M (2006). Global analysis of X-chromosome dosage compensation.. J Biol.

[4] Nguyen DK, Disteche CM (2006). Dosage compensation of the active X chromosome in mammals.. Nat Genet.

[5] Gartler SM, Varadarajan KR, Luo P, Norwood TH, Canfield TK (2006). Abnormal X: autosome ratio, but normal X chromosome inactivation in human triploid cultures.. BMC Genet.

[6] Jacobs PA, Angell RR, Buchanan IM, Hassold TJ, Matsuyama AM (1978). The origin of human triploids.. Ann Hum Genet.

[7] Vogel W, Trautmann T, Horler H, Pentz S (1983). Cytogenetic and biochemical investigations on fibroblast cultures and clones with one and two active X chromosomes of a 69,XXY triploidy.. Hum Genet.

[8] Zaragoza MV, Surti U, Redline RW, Millie E, Chakravarti A (2000). Parental origin and phenotype of triploidy in spontaneous abortions: predominance of diandry and association with the partial hydatidiform mole.. Am J Hum Genet.

[9] Birchler JA, Riddle NC, Auger DL, Veitia RA (2005). Dosage balance in gene regulation: biological implications.. Trends Genet.

[10] Karaman MW, Houck ML, Chemnick LG, Nagpal S, Chawannakul D (2003). Comparative analysis of gene-expression patterns in human and African great ape cultured fibroblasts.. Genome Res.

[11] Johnston CM, Lovell FL, Leongamornlert DA, Stranger BE, Dermitzakis ET (2008). Large-scale population study of human cell lines indicates that dosage compensation is virtually complete.. PLoS Genet.

[12] Straub T, Gilfillan GD, Maier VK, Becker PB (2005). The Drosophila MSL complex activates the transcription of target genes.. Genes Dev.

[13] Ashburner M, Ball CA, Blake JA, Botstein D, Butler H (2000). Gene ontology: tool for the unification of biology. The Gene Ontology Consortium.. Nat Genet.

[14] Carrel L, Willard HF (2005). X-inactivation profile reveals extensive variability in X-linked gene expression in females.. Nature.

[15] Brown CJ, Greally JM (2003). A stain upon the silence: genes escaping X inactivation.. Trends Genet.

[16] Disteche CM, Filippova GN, Tsuchiya KD (2002). Escape from X inactivation.. Cytogenet Genome Res.

[17] Watanabe M, Zinn AR, Page DC, Nishimoto T (1993). Functional equivalence of human X- and Y-encoded isoforms of ribosomal protein S4 consistent with a role in Turner syndrome.. Nat Genet.

[18] Hong S, Cho YW, Yu LR, Yu H, Veenstra TD (2007). Identification of JmjC domain-containing UTX and JMJD3 as histone H3 lysine 27 demethylases.. Proc Natl Acad Sci U S A.

[19] Lan F, Bayliss PE, Rinn JL, Whetstine JR, Wang JK (2007). A histone H3 lysine 27 demethylase regulates animal posterior development.. Nature.

[20] Maroni G, Plaut W (1973). Dosage Compensation in DROSOPHILA MELANOGASTER Triploids. II. Glucose-6-Phosphate Dehydrogenase Activity.. Genetics.

[21] Lin H, Gupta V, Vermilyea MD, Falciani F, Lee JT (2007). Dosage compensation in the mouse balances up-regulation and silencing of X-linked genes.. PLoS Biol.

[22] Mizuno H, Okamoto I, Takagi N (2002). Developmental abnormalities in mouse embryos tetrasomic for chromosome 11: apparent similarity to embryos functionally disomic for the x chromosome.. Genes Genet Syst.

[23] Zvetkova I, Apedaile A, Ramsahoye B, Mermoud JE, Crompton LA (2005). Global hypomethylation of the genome in XX embryonic stem cells.. Nat Genet.

[24] Fryns JP, van de Kerckhove A, Goddeeris P, van den Berghe H (1977). Unusually long survival in a case of full triploidy of maternal origin.. Hum Genet.

[25] Monkhorst K, Jonkers I, Rentmeester E, Grosveld F, Gribnau J (2008). X inactivation counting and choice is a stochastic process: evidence for involvement of an X-linked activator.. Cell.

[26] Karolchik D, Kuhn RM, Baertsch R, Barber GP, Clawson H (2008). The UCSC Genome Browser Database: 2008 update.. Nucleic Acids Res.

