# Supplementary figures and images for: Dosage Regulation of the Active X Chromosome in Human Triploid Cells
Source: PLoS Genet. 2009 Dec 4;5(12):e1000751. doi: 10.1371/journal.pgen.1000751 (PMC2777382; doi:10.1371/journal.pgen.1000751)

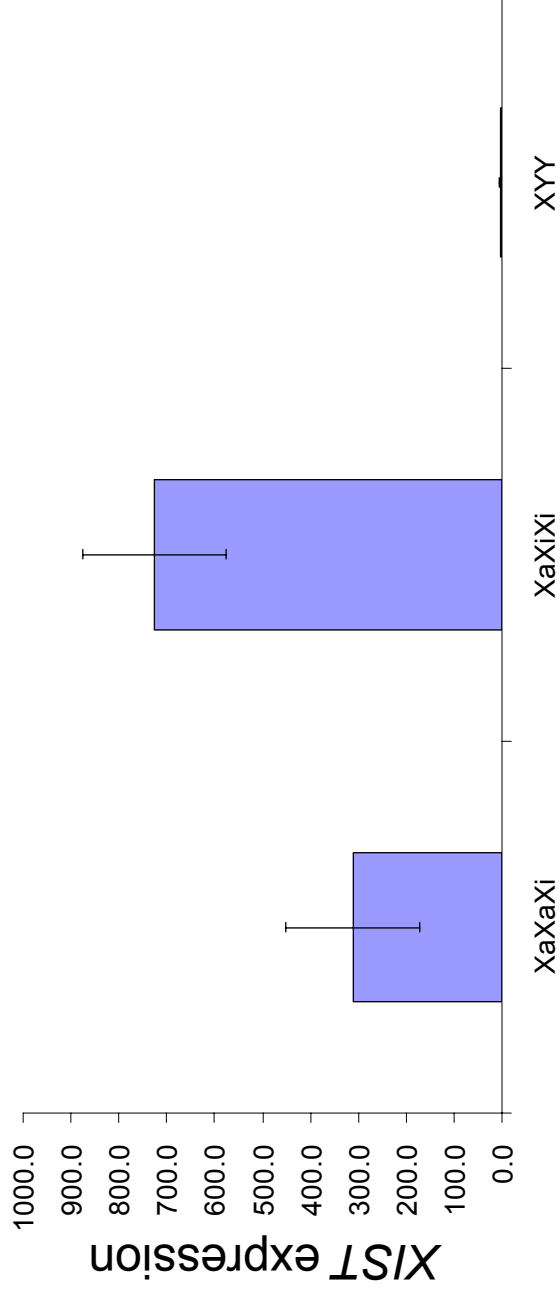

Supplement: Figure S1 — XIST is highly expressed in XXX human triploid fibroblasts but is not expressed in XYY triploid fibroblasts. Expression of XIST is ∼2-fold higher in XaXiXi versus XaXaXi cultures (p = 0.02, student's t-test). Three independent cultures were analyzed for each genotype by expression microarrays. Averages and standard deviations are shown. (0.04 MB PDF) [file pgen.1000751.s001.pdf]

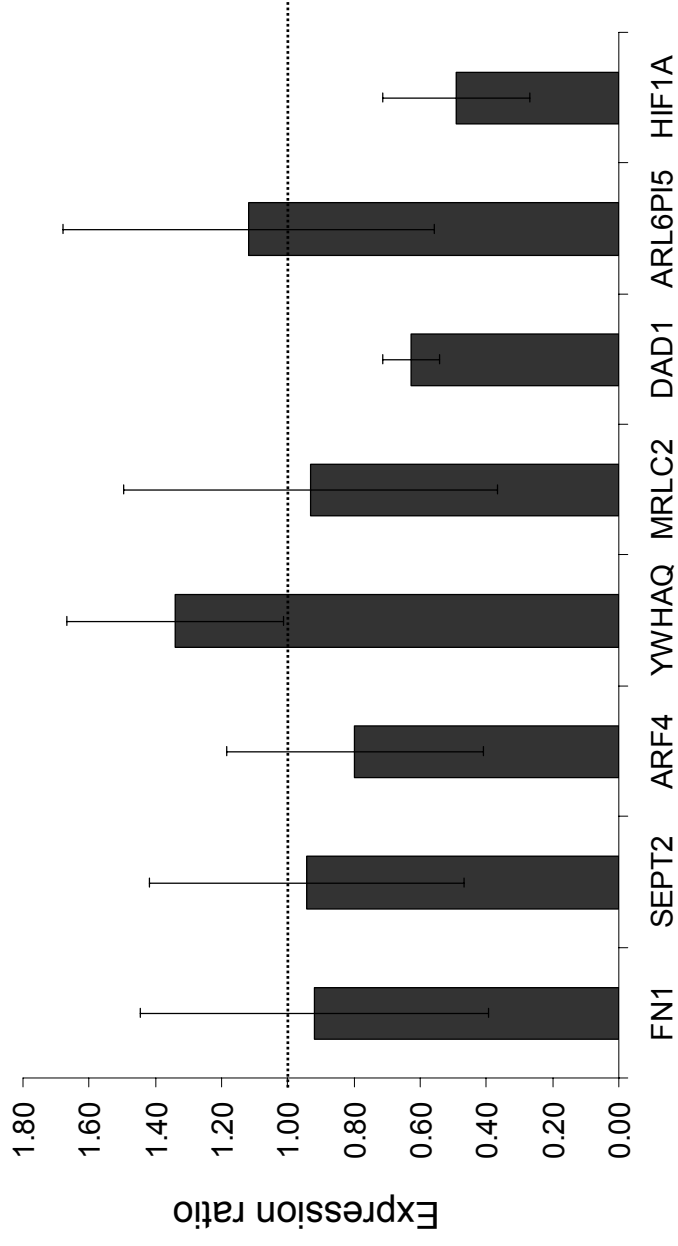

Supplement: Figure S2 — Absolute expression levels of eight autosomal genes are similar in triploid and diploid cultures. The absolute expression level of each gene in triploid cell cultures is represented as the ratio between absolute expression levels in triploid and diploid cultures (mean±standard deviation). The mean fold change between triploid and diploid cultures was 0.90±0.35. Two triploid cultures were analyzed against four female diploid cultures.FN1: fibronectin 1; SEPT2: septin 2; ARF4: ADP-ribosylation factor 4; YWHAQ: tyrosine 3-monooxygenase/tryptophan 5-monooxygenase activation protein, theta polypeptide; MRLC2: myosin regulatory light chain; DAD1: defender against cell death 1; ARL6IP5: ADP-ribosylation-like factor 6 interacting protein 5; HIF1A: hypoxia-inducible factor 1, alpha subunit. (0.04 MB PDF) [file pgen.1000751.s002.pdf]

**A**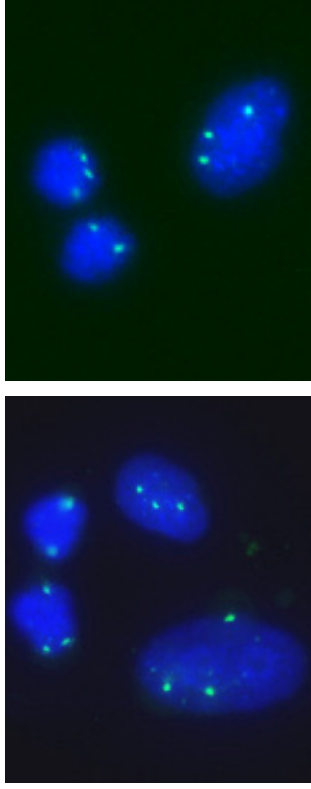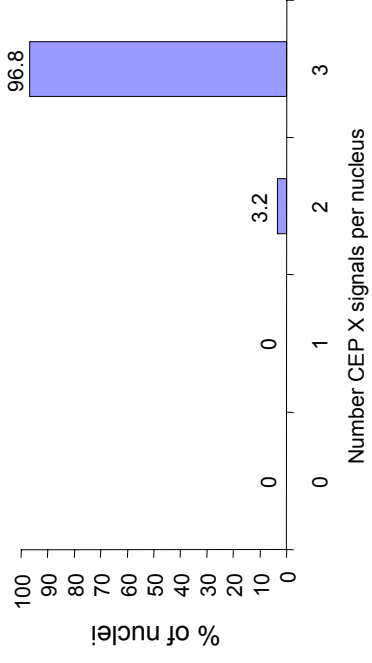**B**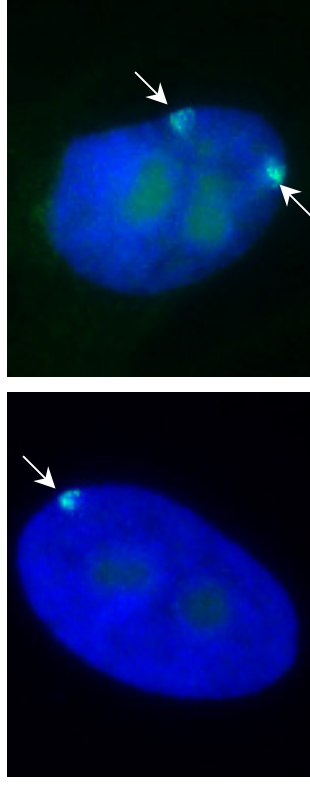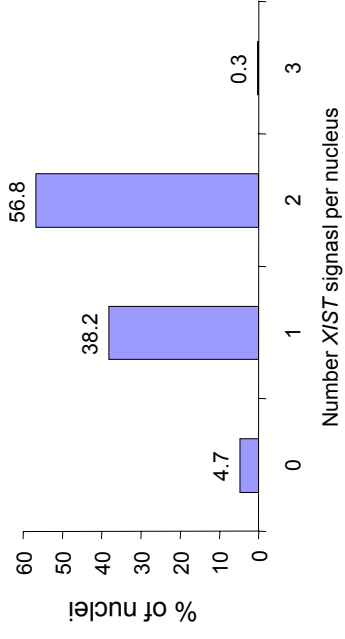

Supplement: Figure S3 — Characterization of the XXXCD2 triploid fibroblasts. (A) Left two panels: examples of DAPI-stained interphase nuclei in which the number of X chromosomes was determined using DNA FISH with an X-linked alpha centrometric (CEP X) probe. Right panel: percentage of 216 nuclei with 0, 1, 2, or 3 signals. (B) Left two panels: examples of DAPI-stained interphase nuclei in which the number of inactive X chromosomes (Xi) was determined by XIST RNA FISH. One nuclei has a single XIST RNA signal and the other has two signals (arrows). Right panel: percentage of 296 nuclei with 0, 1, 2, or 3 XIST RNA signals. (0.08 MB PDF) [file pgen.1000751.s003.pdf]

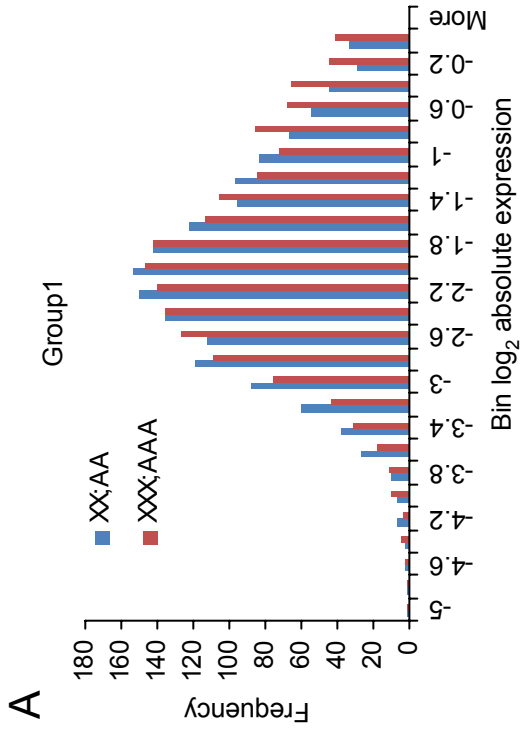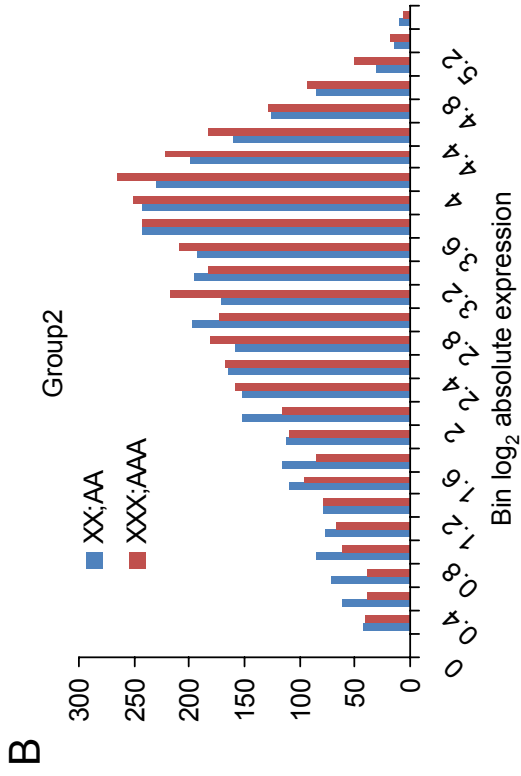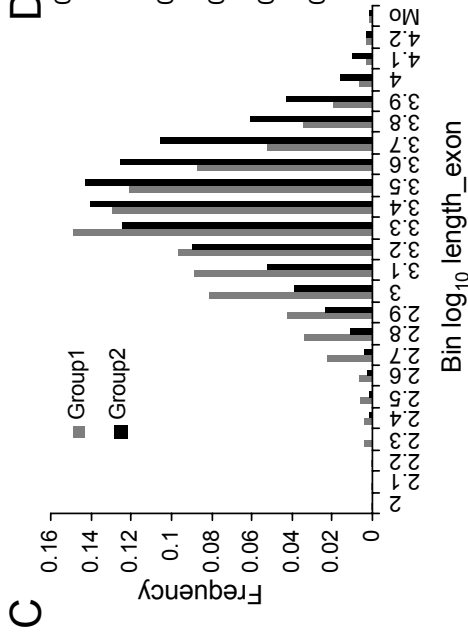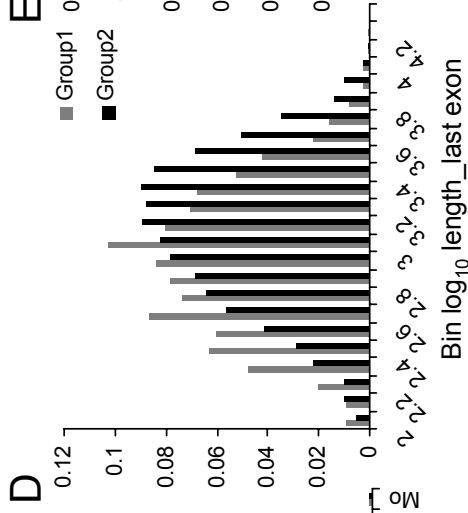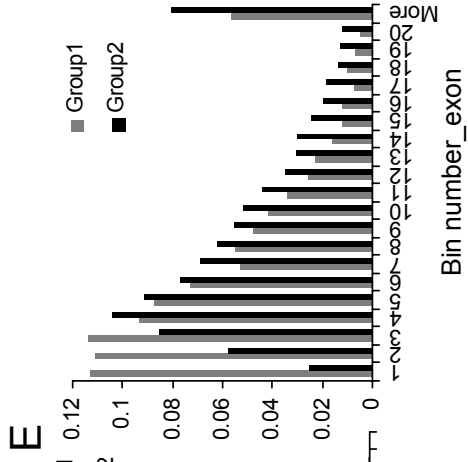

Supplement: Figure S4 — Analyses of autosomal genes showing bimodal expression distribution detected on tiling arrays. Genes were grouped according to their absolute expression level (cDNA/gDNA): <1 (group 1, 1682 genes); ≥1 (group 2, 3,478 genes). (A,B) Distributions of absolute expression of group 1 genes (A) and group 2 genes (B) were similar in diploid (blue bar) and triploid cells (red bar). Absolute expression levels were calculated by averaging cDNA/gDNA ratios (CGH algorithm) of five exon-associated probes at the 3′ end of each gene. Levels were transformed into log2 and binned before graphing the data. (C-E) Compared to group 1 genes (gray bar), group 2 genes (black bar) have longer total-exon-length (C), longer last-exon (D) and more exons per gene (E). Distributions of frequencies of each feature are shown after transformation in log10 and binning. (0.05 MB PDF) [file pgen.1000751.s004.pdf]

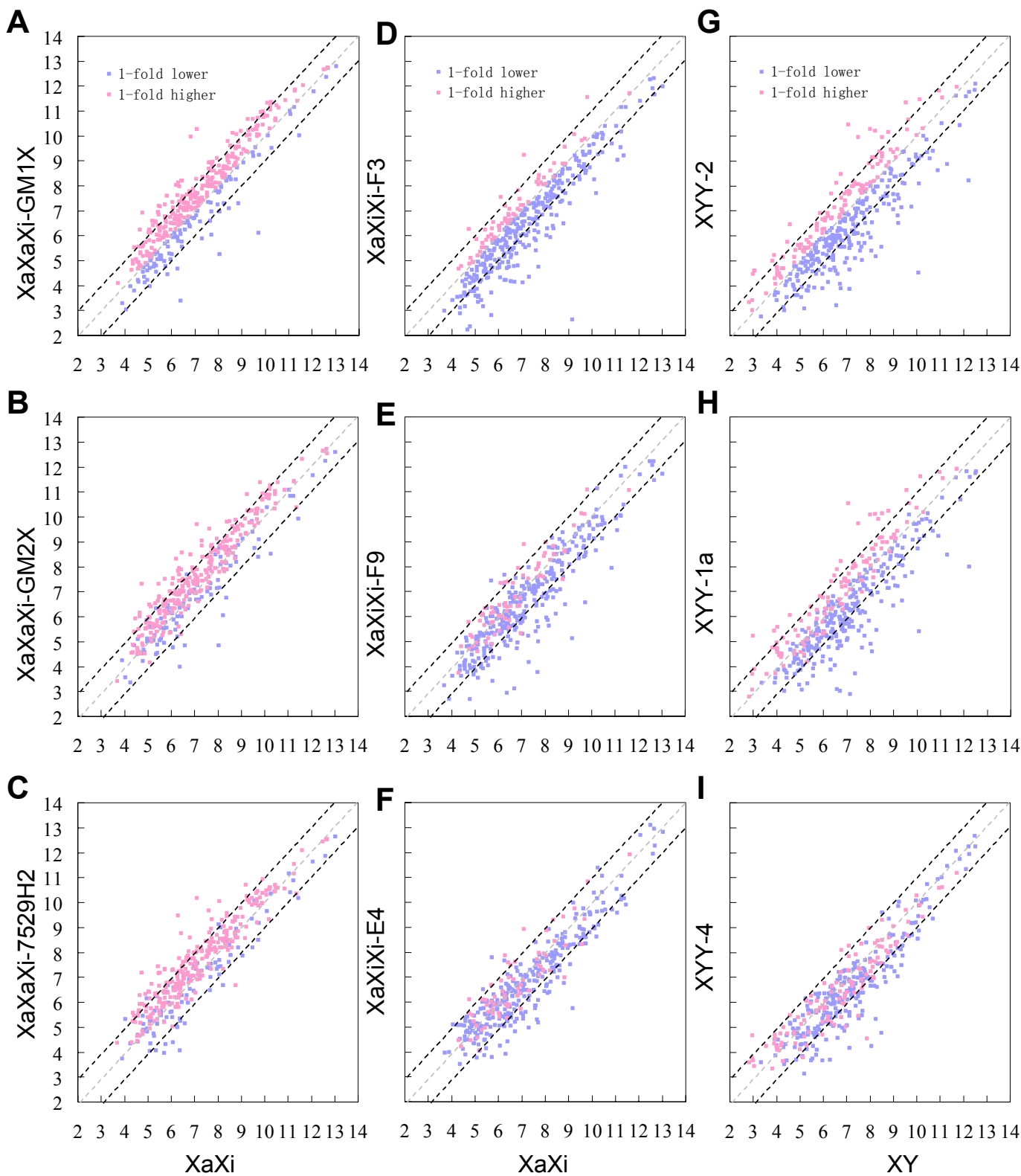

Supplement: Figure S5 — Consistent expression of X-linked genes in individual triploid versus diploid cultures. (A-C) Expression of 69–73% X-linked genes was consistently higher in three individual XaXaXi triploid cultures (GM1X, GM2X, 7529H2) compared diploid XaXi cultures. For comparison the same genes labeled in (A) were also labeled with the same color in (B,C). (D–F) Expression of 71–81% X-linked genes was consistently lower in three individual XaXiXi triploid cultures (F3, F9, E4) versus diploid cultures (XaXi). For comparison the same genes labeled in D were also labeled with the same color in (E,F). (G–I) Expression of 68–71% X-linked genes was lower in three individual XYY triploid cultures (2, 1a, 4) versus diploid XY cultures. For comparison the same genes labeled in (G) were also labeled with the same color in (H) and (I). Scatter plots show expression values (in log2 scale) for 362 X-linked genes after normalized to autosomal gene expression. Pink symbols indicate genes with higher expression and light blue symbols, lower expression. The dotted lines represent two-fold (black), one-fold (gray), and 0.5-fold (black) cutoffs, respectively. (0.14 MB PDF) [file pgen.1000751.s005.pdf]
