# Supplementary material for: Dosage Regulation of the Active X Chromosome in Human Triploid Cells
Source: PLoS Genet. 2009 Dec 4;5(12):e1000751. doi: 10.1371/journal.pgen.1000751 (PMC2777382; doi:10.1371/journal.pgen.1000751)
Supplement: Table S1 — Gene expression in human triploid and diploid fibroblast cultures based on microarray analyses. a Xa: active X chromosome; Xi: inactive X chromosome. b Almost all 111 Y-linked probe-sets included in the human U133 2.0+ Affymetrix arrays showed an Affymetrix “absent call” in female fibroblasts, suggesting no obvious cross-hybridization. In contrast, about 20% of these probe-sets showed a “present call” in male fibroblasts, indicating expression of some Y-linked genes. c The mean signal value for Y-linked probe-sets with Affymetrix “absent call” was used as background level to filter out unexpressed genes on each array. d X:A expression ratios calculated from 16,984 autosomal and 550 X-linked expressed probe-sets for each array. e X:A expression ratios calculated using a previously described method [3]. (0.03 MB DOC) [file pgen.1000751.s006.doc]

| Culture | Triploid fibroblasts | | | | | | | | | Diploid fibroblasts | | | |
| --- | --- | --- | --- | --- | --- | --- | --- | --- | --- | --- | --- | --- | --- |
|  | 75-29-H2 | GM  04939-2X-1 | GM  04939-2X-2 | 75-29-E4 | 75-29-F3 | 75-29-F9 | XYY1 | XYY2 | XYY4 | XX5 | XX6 | XY1 | XY2 |
| Sex chromo. | XaXaXi^a^ | | | XaXiXi | | | XYY | | | XaXi | | XY | |
| Autosome | AAA | | | AAA | | | AAA | | | AA | | AA | |
| Number of arrays | 1 | 1 | 1 | 1 | 1 | 1 | 1 | 1 | 1 | 1 | 3 | 1 | 1 |
| Number of absent Y-probe-sets^b^ | 106 | 103 | 106 | 107 | 102 | 108 | 80 | 80 | 88 | 109 | 103 | 86 | 83 |
| Mean signal value for absent Y-probe-sets^c^ | 11.2 | 7.4 | 11.6 | 7.5 | 13.0 | 19.2 | 20.1 | 20.3 | 31.3 | 9.0 | 11.7 | 8.2 | 24.0 |
| X:A expression ratio^d^ | 1.32 | 1.40 | 1.36 | 0.88 | 0.80 | 0.85 | 0.83 | 0.83 | 0.79 | 1.14 | 1.13 | 1.05 | 1.03 |
| X:A expression ratio^e^ | 1.32 | 1.41 | 1.37 | 0.85 | 0.79 | 0.86 | 0.84 | 0.86 | 0.80 | 1.21 | 1.14 | 1.03 | 1.05 |
