# Supplementary material for: Dosage Regulation of the Active X Chromosome in Human Triploid Cells
Source: PLoS Genet. 2009 Dec 4;5(12):e1000751. doi: 10.1371/journal.pgen.1000751 (PMC2777382; doi:10.1371/journal.pgen.1000751)
Supplement: Table S3 — Primers used for quantitative PCR assays of autosomal genes. a The primer position in the transcript follows the gene symbol (F, forward; R, reverse). b The PCR efficiency and the square of the correlation coefficient (R2) were derived from standard curves. (0.03 MB DOC) [file pgen.1000751.s008.doc]

| Primer | Sequence | Amplicon size | Efficiency ^b^ | R^2, b^ |
| --- | --- | --- | --- | --- |
| ACTG1-F3506^a^ | ACCTGGCATCTTACACGGTC | 96bp | 1.21 | 0.997 |
| ACTG1R3411 | GAACTAGCAAAGCCCACAGC |  |  |  |
| FN1-F6717 | AAGGTTCGGGAAGAGGTTGT | 72bp | 1.02 | 0.998 |
| FN1-R6788 | CGAGTCATCCGTAGGTTGGT |  |  |  |
| SEP2-F545 | GTGGTCGGTGAATCAGGTCT | 176bp | 0.92 | 0.998 |
| SEP2-R720 | ACCACTGTCAGGCGTAGCTT |  |  |  |
| ARF4-F606 | TGCTATGGCCATCAGTGAAA | 165bp | 1.17 | 1 |
| ARF4-R870 | TATTCTGCCCAAACCAGTCC |  |  |  |
| YWHAQ-F315 | GAGCAGAAGACCGACACCTC | 206bp | 0.98 | 0.995 |
| YWHAQ-R520 | CACGCAACTTCAGCAAGGTA |  |  |  |
| MRLC2-F246 | CAGAGATGGCTTCATCGACA | 134bp | 1.09 | 0.993 |
| MRLC2-R379 | TGGTCAGGAACATGGTGAAA |  |  |  |
| DAD1-F125 | GTACTTGAGCTCCACTCCGC | 210bp | 0.93 | 0.998 |
| DAD1-R334 | AAATCCGCTTTGTTCTGTGG |  |  |  |
| ARL6IP5-F421 | GGTCATGTTGGCGAGCTATT | 200bp | 1.11 | 0.992 |
| ARL6IP5-R620 | GTTCTAGGGCATCCAGGACA |  |  |  |
| HIF1A-F695 | TCCATGTGACCATGAGGAAA | 251bp | 0.93 | 0.998 |
| HIF1A-R945 | CCAAGCAGGTCATAGGTGGT |  |  |  |
